# Molecular Diplomacy of Lipids in the War of Immunity: Bridging Rare and Common Disease Mechanisms

**DOI:** 10.3390/ijms26125568

**Published:** 2025-06-10

**Authors:** Manoj Kumar Pandey

**Affiliations:** 1 Division of Human Genetics, Cincinnati Children’s Hospital Medical Center, Cincinnati, OH 45229, USA; manoj.pandey@cchmc.org; 2 Department of Pediatrics, College of Medicine, University of Cincinnati, Cincinnati, OH 45229, USA

## 1. Introduction

The panorama of biomedical and translational research is experiencing a profound conceptual evolution, one that calls for a reassessment of traditional binaries, such as active versus inactive, genetic versus epigenetic, and structural versus metabolic. The five studies highlighted in this editorial exemplify this paradigm shift, each interrogating established assumptions about immune function and biological regulation within its unique context. Yet, despite their disciplinary differences, these investigations converge on a shared revelation that biological systems are not merely collections of isolated pathways or inert components but are highly dynamic and integrative networks capable of delicate responses to internal and external cues. This growing recognition invites a more holistic understanding of biology, one that embraces complexity and redefines what it means for a molecule, structure, or process to be considered “active” in shaping health and disease.

The study by Zhao et al. on polyethylene glycol 400 (PEG400) revolutionizes how pharmaceutical excipients are viewed, showing that this once-thought-inert substance actively modulates hepatic metabolism and nuclear receptor signaling [1]. This finding reopens critical questions about drug formulation safety, especially under chronic exposure, and suggests that excipients may play unrecognized roles in modulating therapeutic outcomes.

Simultaneously, Duan et al. spotlight the epigenetic regulator methyltransferase 3 (METTL3) as a keystone in livestock immunity, revealing how its modulation of N6-methyladenosine (m6A) methylation influences inflammatory and oxidative stress responses in intestinal epithelial cells [2]. The translational potential of this work is profound, offering genetic and biotechnological tools for breeding disease-resistant livestock in an era increasingly focused on sustainable agriculture.

In a parallel exploration of lipid metabolism, Du et al. dissect the molecular synergy between DNA methylation and the Hippo signaling pathway, laying the groundwork for a regulatory model where gene accessibility and signal transduction co-govern metabolic health. Their synthesis offers not only mechanistic depth but also clinical foresight, suggesting novel biomarkers and therapeutic targets in metabolic and oncologic diseases [3].

This metabolic–immunologic nexus finds further reinforcement in Magnusen and Pande’s recharacterization of Fabry disease, not merely as a lysosomal storage disorder but as an immune-mediated inflammatory condition driven by complement system dysregulation [4]. This reframing illustrates new therapeutic avenues, particularly complement-targeted therapies, and reframes clinical strategies for this and similar metabolic disorders.

Finally, Singh et al. shifts the spotlight to one of the most daunting challenges in oncology, which is the cancer stem cells. Their review identifies lipid metabolism as a central enabler of cancer stem cell survival and resistance, offering a compelling argument for targeting this metabolic axis to erode therapy-resistant tumor reservoirs. The integration of lipid modulation with autophagy inhibition and immunotherapy could mark a turning point in durable cancer remission strategies [5].

Each of these studies moves beyond disciplinary silos, instead drawing connections across metabolism, immunity, signaling, and gene regulation. Collectively, they challenge us to reconsider the roles of overlooked agents whether excipients, epigenetic marks, or immune molecules not as inert or peripheral but as active and often decisive determinants of health and disease. In embracing this complexity, we stand to not only refine our scientific models but also to transform clinical and agricultural practices in ways that are more effective, precise, and sustainable.

In this issue, we spotlight a diverse array of following studies that collectively challenge traditional paradigms in pharmacology, metabolism, immunology, and oncology. At the heart of these investigations, there is a common theme: that biological actors, whether traditionally regarded as inert or newly implicated in cross-regulatory networks, can exert significant and often underestimated influence on health and disease.

**A. Uncovering the metabolic influence of PEG400 on hepatic function**

In the long-standing paradigm of pharmaceutical science, excipients have traditionally been regarded as pharmacologically inert vehicles that carry active ingredients without exerting biological effects of their own [6,7]. However, the compelling study by Zhao et al. challenges this foundational assumption and opens an urgent dialog on the functional biology of drug formulation components [1].

The authors employ state-of-the-art proteomics, bioinformatics, and molecular docking techniques to unravel the profound impact of polyethylene glycol 400 (PEG400), which is a widely used pharmaceutical excipient on hepatic metabolism in rodents. Far from being biologically passive, PEG400 was found to significantly modulate the expression of over 150 proteins, particularly those involved in lipid metabolism, retinol processing, bile acid synthesis, and nuclear receptor signaling. These findings underscore a transformational shift in our understanding of how excipients may interact with metabolic pathways and influence therapeutic outcomes.

Notably, the study reveals that PEG400 stimulates the conversion of retinol to retinoic acid (RA), elevates bile acid synthesis, and activates critical nuclear receptors, such as peroxisome proliferator-activated receptor alpha (PPARα), retinoid X receptor alpha (RXRα), and pregnane X receptor (PXR). These changes collectively suggest that PEG400 not only impacts the metabolic fate of co-administered drugs but also possesses intrinsic biological activity capable of modifying endogenous physiological states. The observed upregulation of UDP-glucuronosyltransferase 1a9 (Ugt1a9), which is a key Phase II metabolic enzyme, and the activation of the PPARα-signaling pathway provide strong mechanistic evidence linking PEG400 exposure to enhanced lipid catabolism and altered drug metabolism.

Perhaps most provocative is the suggestion that PEG400 might directly bind and activate nuclear receptors. While traditionally thought of as a passive delivery vehicle, PEG400 here behaves more like a bioactive compound, affecting the transcription of genes involved in metabolism, detoxification, and transport. This raises fundamental questions about the role of excipients in pharmacokinetics and pharmacodynamics and signals a need for regulatory frameworks to adapt to this new understanding.

The implications of this study are both broad and significant. First, it invites a reevaluation of PEG400’s safety profile, particularly in chronic dosing scenarios or in vulnerable populations with altered hepatic function. Second, the study provides a rationale for exploring PEG400 as a potential therapeutic adjunct in metabolic disorders, such as obesity and dyslipidemia, given its apparent activation of fat-oxidation pathways and attenuation of lipid accumulation. Finally, these insights could transform formulation science by positioning excipients not just as passive ingredients but as active participants in therapeutic design.

Zhao et al. have delivered a timely and rigorously executed study that challenges assumptions, provides mechanistic clarity, and opens new avenues for both basic and translational research. As the boundaries between excipients and actives blur, we are reminded that, in biology, few substances are truly inert. It is time for the pharmaceutical sciences to fully embrace the complexity and potential of the so-called “inactive” ingredients.

**B. Epigenetic regulator methyltransferase 3 (METTL3) at the crossroads of immunity and genetic resilience**

The livestock industry is constantly challenged by the vulnerability of neonatal animals to infections, particularly those that compromise gastrointestinal health [8,9,10]. In their thought-provoking study, Duan et al. illuminated a promising new avenue in disease resistance research by exploring the epigenetic regulator methyltransferase 3 (METTL3) and its role in intestinal inflammation, apoptosis, and oxidative stress [2].

This research investigates a compelling intersection of immunogenetics and molecular epigenetics, revealing how N6-methyladenosine (m6A) modification, particularly catalyzed by METTL3, influences the response of intestinal epithelial cells (IECs) to bacterial endotoxins. The study highlights the dramatic impact of lipopolysaccharide (LPS) exposure on IECs mimicking bacterial infection and uncovers METTL3 as a pivotal player in mediating the cellular damage that ensues.

The authors systematically demonstrate that overexpression of METTL3 amplifies inflammatory cytokines (e.g., IL1β, IL6, and TNFα) expression, promotes apoptosis, and increases reactive oxygen species (ROS) production. In contrast, METTL3 silencing mitigates these effects, thereby safeguarding cellular viability and restoring redox balance. These findings suggest that METTL3 is not just a passive epigenetic writer but a dynamic regulator of immune and stress responses, with implications that extend beyond basic biology into the realm of genetic selection and breeding.

What sets this study apart is its translational potential. As METTL3 expression appears to sensitize IECs to LPS-induced injury, targeting METTL3 may represent a novel genetic strategy for enhancing disease resistance in sheep. This is especially relevant in the context of marker-assisted selection (MAS) or more cutting-edge gene-editing approaches, which are rapidly gaining traction in livestock improvement programs.

Importantly, this study is among the first to characterize the functional role of m6A modifications in the immune responses of sheep epithelial cells, expanding the scope of mammalian epigenetics into a species that is often underrepresented in molecular immunology research. It also underscores the role of epigenetic plasticity in shaping innate immunity, offering exciting possibilities for fine-tuning host resilience without compromising physiological functions.

However, as the authors rightly note, translating these findings into breeding programs will require further investigation, including large-scale population studies to identify favorable METTL3 alleles and a deeper understanding of the regulatory elements of the gene. Functional genomics tools such as CRISPR/Cas systems and emerging gene delivery platforms like IS-Dra2-TnpB offer the technical means to move from associative studies to causal validation and trait enhancement.

This work by Duan et al. reinforces a growing consensus in the field: epigenetic factors are not just peripheral modifiers but central to disease susceptibility and resilience. The METTL3-m6A axis may well become a cornerstone in next-generation livestock-breeding strategies that combine genetic precision with immune robustness.

In a time when the demands on food security and sustainable animal health are more pressing than ever, this study offers a clear message that the future of disease resistance in livestock lies in understanding and harnessing the biology of epigenetic regulation.

**C. Epigenetic marks and signaling pathways orchestrate lipid dynamics**

In the rapidly evolving field of metabolic research, the interplay between epigenetic mechanisms and intracellular signaling pathways is emerging as a cornerstone of disease understanding and therapeutic innovation [11,12,13]. The paper by Du et al. offers a timely and insightful synthesis of two seemingly distinct yet functionally intertwined biological processes, 5-methylcytosine (5mC) DNA methylation and the Hippo signaling pathway, and their shared governance over lipid homeostasis [3].

Lipid metabolism, a tightly regulated process central to energy balance, inflammation, and cellular structure, has long been studied in the context of enzyme activity and gene expression. What this review eloquently underscores is the need to look deeper into the regulatory architecture that dictates gene accessibility and cellular responsiveness. In this light, 5mC methylation emerges as a master switch, capable of silencing or activating genes involved in lipid synthesis, transport, and degradation.

The authors also cast a much-needed light on the Hippo pathway, known for its role in organ size control and tumor suppression, as a critical regulator of adipocyte differentiation and lipid storage. The novel proposition explored here is the molecular crosstalk between 5mC methylation and Hippo signaling effectors, such as YAP/TAZ. Though still in the early stages of elucidation, this interaction hints at a complex, multilayered control system where signaling inputs and epigenetic states converge to fine-tune metabolic outcomes.

This is more than theoretical speculation. The potential for clinical translation is profound. The review rightly outlines how combining methylation profiling with assessments of Hippo pathway activity could yield biomarkers for early diagnosis and risk prediction in diseases like obesity, fatty liver, and cardiovascular disorders. Moreover, the possibility of manipulating this regulatory axis opens new doors for personalized interventions and novel drug targets, not only for metabolic diseases but also for malignancies where lipid metabolism is co-opted by tumor cells.

Future research will need to address several key questions, such as the following: How do these two systems interact across different cell types? Are their interactions direct or mediated through intermediates like chromatin remodelers or non-coding RNAs? Can dynamic changes in 5mC marks and Hippo signaling be leveraged for real-time disease monitoring or adaptive therapeutic strategies?

Du et al. present a persuasive argument that integrating epigenetics with signal transduction is not just a conceptual innovation; it is a necessary evolution in our approach to metabolism. Their work should inspire a new wave of interdisciplinary research aimed at deciphering this regulatory nexus at both molecular and systemic levels.

The synergistic regulation of lipid metabolism by 5mC methylation and the Hippo pathway is not merely a hypothesis to be tested; it is a paradigm waiting to be realized.

**D. Lipid-induced complement activation in rare genetic diseases**

The complement system is a crucial part of the innate immune response, consisting of a cascade of plasma proteins that enhance the ability of antibodies and phagocytic cells to clear pathogens [14]. One of its key functions involves the production of small peptide fragments, such as C3a and C5a, which are generated during the activation of the complement pathways, such as classical, lectin, or alternative. When C3 is cleaved by C3 convertase, it produces C3a and C3b; similarly, cleavage of C5-by-C5 convertase yields C5a and C5b. C3a and C5a act as potent anaphylatoxins, meaning they promote inflammation by increasing vascular permeability, inducing smooth muscle contraction, and recruiting immune cells, such as monocytes, macrophages, dendritic cells, and neutrophils, to sites of infection or injury [15]. C5a is one of the most powerful chemotactic agents known, guiding immune cells toward the site of complement activation. Together, C3a and C5a help amplify the immune response and facilitate rapid clearance of pathogens [14,15,16]. However, when these anaphylatoxins are generated in excessive or uncontrolled amounts, they can contribute to pathological inflammation [17,18,19]. C3a and C5a bind to their respective receptors on immune cells, i.e., monocytes, macrophages, dendritic cells, mast cells, neutrophils, and endothelial cells, triggering the release of pro-inflammatory cytokines such as IFNg, TNF α, IL1β, IL6, IL12, and IL17, which leads to vasodilation, increased vascular permeability, and leukocyte infiltration, which are all hallmarks of tissue inflammation [20,21,22,23].

In diseases such as systemic lupus erythematosus (SLE), rheumatoid arthritis (RA), and age-related macular degeneration (AMD), chronic complement activation has been implicated in sustaining inflammation and causing tissue damage. For example, in RA, C5a attracts neutrophils into the synovial joints, where they release enzymes and reactive oxygen species that degrade cartilage and bone [24,25,26]. Similarly, in SLE, deposition of immune complexes triggers complement activation, causing a release of C3a and C5a, which recruit inflammatory immune cells and damage tissues such as the kidneys (lupus nephritis) [27,28]. Elevated levels of C3a and C5a have been linked to the development of age-related macular degeneration [29,30,31]. Moreover, in sepsis, hypoxia, or acute respiratory distress syndrome (ARDS), overwhelming C5a production can lead to systemic inflammation, vascular leakage, and multi-organ failure [32,33,34]. These examples feature how the dysregulation of complement-derived inflammatory mediators can transform a protective response into a driver of chronic or acute pathological inflammation.

The complement system is increasingly recognized as a critical mediator of tissue inflammation in various lysosomal storage diseases (LSDs), where persistent immune activation contributes to progressive tissue damage. In these disorders, the accumulation of undegraded substrates not only disrupts cellular function but also appears to provoke a sustained inflammatory response, in part through complement activation.

In Gaucher disease, a lysosomal storage disorder resulting from mutations in the GBA1 gene deficiency in the enzyme glucocerebrosidase leads to the pathological accumulation of glucosylceramide (GC) within cells. This buildup has been associated with elevated tissue expression of complement components C3 and C5. Furthermore, activation of the complement 5a (C5a)–C5aR1 receptor axis has been implicated in driving the increased production of pro-inflammatory cytokines, contributing to the chronic inflammatory environment observed in the disease [35]. Similarly, in Niemann–Pick disease type C, activation of early complement components, such as C1q and C3, has been linked to neuroinflammation and microglial cell activation [35]. In a reported case of Pompe disease, secondary membranous nephropathy following enzyme replacement therapy (ERT) was accompanied by subepithelial deposition of C3 and the membrane attack complex C5b-9 [36].

These observations suggest that complement dysregulation may be a shared pathogenic feature across multiple LSDs. However, the precise mechanisms by which complement activation is initiated and perpetuated and how it contributes to chronic innate immune activation and tissue injury have remained poorly understood.

Our studies have addressed this gap in the context of Gaucher disease, where we identified a critical role for GC-specific IgG autoantibody-mediated complement activation, resulting in robust C5a generation. Importantly, C5a engagement with its receptor, C5aR1, triggers a pro-inflammatory cascade that includes upregulation of UDP-glucose ceramide glucosyltransferase (UGCG), a key enzyme in GC biosynthesis creating a pathogenic feed-forward loop.

GC accumulation in macrophages and dendritic cells leads to the formation of GC-specific IgG immune complexes, which further amplify complement activation and C5a production. Binding of C5a to C5aR1 drives a potent inflammatory response, marked by innate and adaptive immune cells activation and increased production of cytokines such as IFN γ, TNFα, IL1β, IL6, IL12, and IL17. This signaling also promotes the recruitment and activation of macrophages, dendritic cells, and T cells, thereby sustaining a chronic inflammatory environment that contributes to progressive organ damage in Gaucher disease.

Using both Gba1 prone experimental mouse models and human cell-based systems of Gaucher disease, we have also demonstrated that genetic ablation or pharmacological inhibition of C5aR1 significantly reduces inflammatory responses and provide protection against tissue damage. These findings not only clarify the role of complement in Gaucher disease but also point to the C5a–C5aR1 axis as a promising therapeutic target for managing inflammation in LSDs more broadly [37,38,39].

In the compelling detailed review by Magnusen and Pandey, the authors present here a transformative lens through which to view Fabry disease, one that expands the focus from metabolic insufficiency to immunological dysregulation. This shift is not merely academic; it has profound implications for both our understanding of Fabry pathogenesis and the design of future therapeutic strategies [4].

Fabry disease, long classified as a lysosomal storage disorder due to its hallmark accumulation of globotriaosylceramide (Gb3), is re-envisioned here as an immunologically active condition. The review outlines how this lipid storage anomaly does not remain confined within lysosomes but instead sparks a systemic inflammatory response via complement activation, particularly through the potent anaphylatoxins C3a and C5a [32,35,40].

This mechanistic exposition is one of the central contributions of the paper. The authors skillfully connect molecular signals to cellular behaviors, showing how C3a and C5a engage their receptors (C3aR and C5aR1) on both leukocytes and endothelial cells, leading to the pathological upregulation of adhesion molecules such as VCAM1, ICAM1, PECAM1, and CR3. This cascade facilitates the excessive recruitment and transmigration of immune cells, a process that turns a defense mechanism into a driver of chronic inflammation and tissue injury.

Particularly insightful is the link the authors draw between complement activation and the formation of immune complexes, either from accumulated Gb3 or anti-drug IgG in patients receiving enzyme replacement therapy. This framing elegantly reconciles clinical observations with immunopathological theory, suggesting why enzyme replacement therapy, though essential, may inadvertently worsen inflammatory signaling in some patients. This nuanced understanding of immune complex formation as a double-edged sword offers an urgent call to re-evaluate long-term therapeutic strategies.

Another compelling element is the discussion of endothelial dysfunction, which is presented not as a downstream effect of Fabry pathology but as a co-conspirator in disease progression. The authors show how inflammatory cytokines, complement fragments, and immune cell trafficking directly erode the vascular glycocalyx, compounding organ damage. By doing so, they place vascular health at the center of clinical manifestations of Fabry disease from early neuropathies to later renal and cardiac complications.

This review also provides a powerful synthesis of existing and emerging therapeutic avenues. Highlighting agents like eculizumab, pegcetacoplan, and Avacopan, the authors point to the untapped potential of complement-targeted therapies in Fabry disease. The case report of successful eculizumab use in a patient with overlapping Fabry and atypical hemolytic–uremic syndrome underscores the feasibility and urgency of clinical trials in this direction.

However, the article does more than simply recount a litany of molecular interactions. It poses critical, forward-looking questions, such as the following: Could the upregulation of soluble adhesion molecules serve as a diagnostic biomarker or therapeutic target? Might selective complement inhibition offer a tailored approach for Fabry subtypes or treatment-resistant cases? These questions challenge us to not only understand the disease better but also to refine the clinical tools and strategies used to manage it.

In sum, the work performed by Magnusen and Pandey is not just a review; it is reorientation. By elucidating the immunological choreography that underpins Fabry disease, they invite clinicians, researchers, and drug developers to consider the immune system not as a bystander but as a central actor in both pathogenesis and potential treatment. As we move toward precision medicine, this paper marks a pivotal step in reimagining Fabry disease not simply as a lysosomal storage disorder but as a complex immunometabolism condition requiring equally sophisticated interventions.

**E. Targeting lipid metabolism to disarm cancer stem cells and resist relapses**

Cancer stem cells (CSCs) are a subset of tumor cells that drive tumor initiation, progression, metastasis, and recurrence [41]. A range of surface markers, including CD24, CD34, CD44, CD47, CD90, CD133, aldehyde dehydrogenase 1 family member (ALDH1m), epithelial cell adhesion molecule, (EpCAM), leucine-rich repeat-containing G protein-coupled receptor 5 (LGR5), stage-specific embryonic antigen-1 (SSEA-1), epidermal growth factor receptor (EGFR), a receptor tyrosine kinase (KIT), transcription factors, i.e., SOX2, NANOG, POU5F1, or OCT4 have been identified to aid in their detection and isolation across various malignancies [42,43,44,45,46,47,48].

In the ongoing battle against cancer, traditional treatment modalities such as chemotherapy and radiotherapy, and even the more advanced immunotherapies often confront a resilient and elusive adversary in CSCs [49,50,51]. These microscopic survivors have an uncanny ability to evade immune destruction, endure cytotoxic treatments, regenerate tumors, and initiate metastases, undermining long-term treatment success [43,52]. Moreover, the tumor microenvironment (TME) composed of cancer-associated fibroblasts (CAFs), immune cells, adipocytes, endothelial cells, neuroendocrine (NE) cells, extracellular matrix (ECM), and extracellular vesicles plays an essential role in preserving CSC stemness. The TME not only provides physical protection but also secretes signaling molecules that reinforce CSC survival and resistance, shielding them from both the host immune response and therapeutic interventions [53,54,55].

Together, the inherent properties of CSCs and their complex interactions with the TME highlight the urgent need for novel therapeutic strategies. These must be capable of targeting CSCs specifically while also disrupting the supportive niche that enables their persistence and pathogenicity.

The recent review by Singh et al. shines a vital spotlight on the metabolic lifelines that sustain these cells and offers a promising roadmap to finally outmaneuver them. The study draws attention to lipid metabolism as a linchpin in CSC survival and therapy resistance. Far from being passive structural components, lipids in CSCs act as dynamic regulators fueling fatty acid oxidation (FAO), buffering oxidative stress, and shaping mitochondrial function. These metabolic features are not incidental; they are strategic adaptations that empower CSCs to resist therapy, remain dormant, and later reemerge stronger [5].

The intersection of lipid metabolism with autophagy, a process of cellular self-renewal, represents a particularly exciting and underexplored battleground. Singh and colleagues explore how autophagy fine-tunes lipid utilization in CSCs, ensuring metabolic plasticity and survival. Inhibitors targeting enzymes, like fatty acid synthase (FASN), glucosylceramide synthase (GCS), and fatty acid transport protein 2 (FATP2), or autophagy mediators, such as Unc-51-like autophagy-activating kinases 1 (ULK1) and the mammalian target of rapamycin (mTOR), have shown promising results in sensitizing CSCs to standard treatments across multiple cancer types. These findings suggest that disrupting lipid storage and catabolism could cripple the very foundation of CSC resilience.

Importantly, the paper does not shy away from the complexity of this approach. CSCs are not a uniform enemy. Their metabolic preferences, ranging from lipid dependency to glucose oxidation, are as diverse as the tumors they inhabit. This heterogeneity presents both a challenge and a call to precision medicine: one-size-fits-all therapies will not suffice. The authors rightly highlight the need for combinatorial strategies that address multiple metabolic pathways while minimizing toxicity to normal stem cells and tissues.

Moreover, the implications of this research extend beyond killing cancer cells, they touch on redefining cancer as a metabolic disease. The link between lipid metabolism, immune evasion, and autophagy regulation opens the door to synergistic treatment strategies. Combining lipid-modulating agents with immunotherapy or mRNA-based cancer vaccines, as proposed in the paper for melanoma harboring the B-Raf proto-oncogene, serine/threonine kinase (BRAF) V600E mutation, offers a promising avenue to enhance therapeutic efficacy by targeting multiple tumor survival pathways in parallel.

As promising as these insights are, the path forward is steep. Off-target effects, metabolic redundancy, and adaptive resistance remain formidable obstacles. Yet, this review provides a compelling vision and an urgent imperative to disrupt the metabolic sanctuary of CSCs. In doing so, we may finally tilt the balance from disease recurrence to durable remission.

Overall, Singh et al. do more than catalog the metabolic quirks of CSCs; they elevate lipid metabolism from a supporting role to a starring one in the narrative of cancer treatment. By illuminating this metabolic Achilles heel, they offer the field a fresh perspective and a fertile ground for future breakthroughs. The war on cancer may be long, but targeting lipid metabolism in CSCs could be the turning point we have been waiting for.

## 2. Conclusions

Taken together, these five groundbreaking studies illuminate a powerful new narrative in biomedical and translational research, one where the boundaries between passive and active, supportive and central, genetic and metabolic, are increasingly blurred. What once was considered inert, like PEG400, now emerges as a modulator of nuclear receptor signaling and hepatic function. What was presumed to be a peripheral epigenetic mark, such as METTL3-driven m6A modification, is revealed to be a master regulator of immune defense and cellular resilience. Lipid metabolism, traditionally seen as a downstream outcome of genetic programming, is now understood as a dynamic battlefield, shared by cancer stem cells, immune pathways, and developmental signaling axes like Hippo.

This convergence of insights underlines a central truth that biology does not operate in isolated silos but in deeply interconnected networks where even minor components, be they excipients, epigenetic writers, or signaling intermediates, can have profound physiological consequences. The future of medicine, agriculture, and biotechnology lies not in targeting single pathways but in embracing this complexity to engineer systems-level precision and resilience.
